# Motivational determinants among physicians in Lahore, Pakistan

**DOI:** 10.1186/1472-6963-10-201

**Published:** 2010-07-09

**Authors:** Ahmad Azam Malik, Shelby Suzanne Yamamoto, Aurélia Souares, Zeeshan Malik, Rainer Sauerborn

**Affiliations:** 1 Institute of Public Health, University of Heidelberg, Heidelberg, Germany; 2 Mannheim Institute of Public Health, University of Heidelberg, Mannheim, Germany; 3 Sir Ganga Ram Hospital, Lahore, Pakistan

## Abstract

**Introduction:**

Human resource crises in developing countries have been identified as a critical aspect of poor quality and low accessibility in health care. Worker motivation is an important facet of this issue. Specifically, motivation among physicians, who are an important bridge between health systems and patients, should be considered. This study aimed to identify the determinants of job motivation among physicians, a neglected perspective, especially in developing countries.

**Methods:**

A stratified random sample of 360 physicians was selected from public primary, public secondary and public and private tertiary health facilities in the Lahore district, Pakistan. Pretested, semi-structured, self-administered questionnaires were used. For the descriptive part of this study, physicians were asked to report their 5 most important work motivators and demotivators within the context of their current jobs and in general. Responses were coded according to emergent themes and frequencies calculated. Of the 30 factors identified, 10 were classified as intrinsic, 16 as organizational and 4 as socio-cultural.

**Results:**

Intrinsic and socio-cultural factors like serving people, respect and career growth were important motivators. Conversely, demotivators across setups were mostly organizational, especially in current jobs. Among these, less pay was reported the most frequently. Fewer opportunities for higher qualifications was a demotivator among primary and secondary physicians. Less personal safety and poor working conditions were important in the public sector, particularly among female physicians. Among private tertiary physicians financial incentives other than pay and good working conditions were motivators in current jobs. Socio-cultural and intrinsic factors like less personal and social time and the inability to financially support oneself and family were more important among male physicians.

**Conclusion:**

Motivational determinants differed across different levels of care, sectors and genders. Nonetheless, the important motivators across setups in this study were mostly intrinsic and socio-cultural, which are difficult to affect while the demotivators were largely organizational. Many can be addressed even at the facility level such as less personal safety and poor working conditions. Thus, in resource limited settings a good strategic starting point could be small scale changes that may markedly improve physicians' motivation and subsequently the quality of health care.

## Introduction

The workforce is arguably the most important input to any health system and has a strong impact on overall health system performance [1]. According to World Health Organization (WHO), there is a worldwide estimated shortage of 4.3 million health workers, primarily concentrated in south Asia followed by Africa [2]. These areas also suffer the greatest burden of disease, worsened by having to cope with a much smaller health workforce [2]. Sub-Saharan Africa and southeast Asia together have 53% of the global disease burden but only 15% of the world's health care workforce [3].

Compounding this problem are low levels of health care provider (HCP) motivation. It has often been identified as a central problem in this human resource crisis and consequently, health service delivery and quality [4]. Health care delivery is highly labor-intensive, and service quality, efficiency and equity are all directly related to providers' willingness to apply themselves to their tasks. Low motivation leads to the insufficient translation of knowledge, the underutilization of available resources and weak health system performance [5,6].

Motivation is a process that results from the dynamic interactions between individuals, their work environment and communities or society [7]. HCP motivation encompasses determinants that drive performance of a task, independent of the resources and knowledge available. Failure to account for HCP motivation can hamper the development of health care systems. In a rural district of Tanzania, efforts to provide access to facilities and competent clinicians to improve the quality of care was unsuccessful due to a lack of staff motivation [8].

Motivation is not only important for patient satisfaction, productivity, and health care sector performance but also in retaining well-performing staff [9]. Low motivation adds to the push factors for the migration of health providers, both from rural areas to the cities and out of the country [10]. Consequently, a motivated workforce is critical in retaining qualified health staff and the achievement of health services targets and reforms [11].

The health care sector is not only facing problems stemming from the shortage of skilled labor but also the increasing cost and complexity of technology, intensifying demands from the aging population, changing regulations of services for continuous quality improvement, increasing orientation towards consumers and various ongoing reorganizations [12]. In particular, in dense, urban cities in developing countries, these demands and shortages can be magnified. Thus, the challenges with respect to HCP motivation in these areas may require particular consideration.

Importantly, although employee motivation is a significant element of health systems performance, it is largely understudied [7] and little attention has been paid to this issue in developing and poor countries [4]. Not enough is currently known about which determinants of motivation are most important to different cadres of workers in developing countries [7,13,14]. Specifically, even fewer studies have concentrated on physician motivation [15]. Physicians act as the bridge between health systems and patients, play a critical role in the distribution and functioning of health system resources and are major stakeholders in the overall performance of health care organizations and the delivery of quality health care services [16,17]. Therefore, the aim of this descriptive part of the larger study examining various aspects of motivation was to identify the most important motivators and demotivators of physicians in different hospitals and clinical settings in the Lahore district of Pakistan, for the purpose of exploring areas for sustainable and attainable improvement.

## Methods

### Health system in Pakistan

In Pakistan, health services are provided through a three-tiered health care infrastructure and a range of public health intervention programs. The former includes Basic Health Units (BHUs), Rural Health Dispensaries (RHDs) and Rural Health Centers (RHCs) as the major primary healthcare facilities. Secondary care includes first and secondary referral facilities providing acute, ambulatory and inpatient care provided by Tehsil Headquarter Hospitals (THQs) and District Headquarter Hospitals (DHQs). These are supported by tertiary level teaching hospitals. Primary and secondary facilities are public institutions while tertiary are both public and private.

Prior to devolution, the delivery of health services was the responsibility of the provincial governments. With the passage of the Local Government Ordinance in 2001, the responsibility for providing health care was almost entirely delegated to the District Governments, with the exception of the large teaching hospitals and their attached medical or dental colleges, which remained under the direct control of the provincial government.

Pakistan has one of the largest public sector-owned service delivery infrastructures in the world [18]. The majority of health service providers in low and middle income countries report their primary site of employment as the public sector (> 70% of doctors and > 50% of other health service providers). The distinction between public and private sectors in Pakistan is not very clear and many public sector practitioners also practice privately - legally or illegally [18]. Many in this region report receiving a large part of their income directly from patients rather than from the government [2].

Pakistan is 122^nd ^according to the WHO ranking of the world's health systems [19] and currently has 0.73 physicians and 0.31 nurses per 1,000 population [20]. As of 2005, Pakistan had approximately 74,000 practicing physicians and almost 1,700 emigrating every year [21]. According to 'Pakistan Medical and Dental Council' (PMDC) almost 129,229 physicians were registered by April 2010 [22]. In addition, with the annual graduation of approximately 6800 medical graduates [21], the number of younger physicians is expected to increase substantially in the coming years. Importantly, this age group is also more likely to emigrate either from rural areas to urban or to other countries [23,24]. Of the 10,651 general practitioners registered between January 1, 2008 to June 30, 2009, 5,128 were female while 4,228 were male. This trend is even stronger among medical graduates who pass their final examinations. As of the end of 2009, females comprised 39% of all registered physicians [22].

### Study site

The study was conducted in the densely populated district of Lahore (4681 persons/km^2^) in Pakistan [25]. The level of urbanization in Pakistan is the highest in South Asia and its urban population is likely to equal its rural population by 2030 [26]. This trend combined with the current health settings and availability of resources was considered in selecting the study location. The Lahore district includes the second largest city of Pakistan (Lahore) with around 7.1 million people, which includes the suburban areas [27]. It has 37 BHUs, six RHCs and 23 RHDs for primary health care services. Secondary health facilities include two Tehsil and two District Headquarter Hospitals. According to PMDC there are 11 public and 15 private teaching medical institutes with affiliated teaching hospitals in Punjab, the most populous province of Pakistan. Among these four of the public and eight of the private teaching medical institutes are in Lahore, which serve a major part of the province of Punjab [22].

### Study outline

The study consisted of three parts to maximize the information gathered from the participants. Part one of the study consisted of open ended questions about the motivators and demotivators from physicians' perspectives in their own words. The second part consisted of a questionnaire designed using a Likert scale for the quantification and statistical analysis of the factors related to motivation. The third part involved 16 in-depth one-on-one interviews (four interviews at each health facility level were included in the study, with equal male and female participation) for the purpose of building contextual understanding of motivation. The results presented here include physicians' responses to open ended questions regarding their five most important work motivators and demotivators within the context of their current jobs and in general. The description and results of second and third parts of the study will be submitted for publication at a later date.

### Participant selection

**Figure 1:**
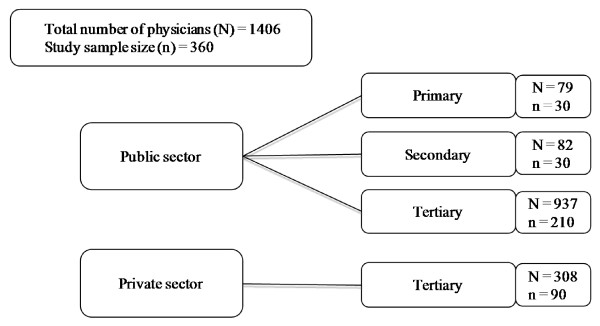
**Sample selection of study participants by strata**.

A stratified random sample of 360 physicians was selected from the four strata (Figure 1). Equal numbers of male and female participants were chosen at each stratum. The health setups represented in the study included all of the public primary and secondary health facilities. The two tertiary facilities (one private and one public) employing the largest number of physicians were also included. All registered medical practitioners from the PMDC working in the study health facilities at the time of recruitment were eligible for the study.

### Questionnaire

Pretesting was completed with 30 physicians in non-study health facilities in Lahore to evaluate and improve the questionnaire. Following the pre-test stage, two experts, each from the field of human resource management, hospital management and psychometrics, reviewed the final instrument. The final questionnaire consisted of semi-structured, self-administered questions. To reduce the possibility of bias, two researchers (AAM, SSY) worked in parallel during data analysis, coding and theme identification. Key themes from the responses were coded after analysis according to which factors (motivators and demotivators) were identified and frequencies calculated. Factors were subsequently categorized as intrinsic, organizational or socio-cultural by three independent researchers (AAM, AS, SSY). In the case of differences, categorizations were discussed until consensus was reached.

### Ethical considerations

Written informed consent was obtained from all of the study participants. Ethical approval was received both from the University of Heidelberg, Medical Faculty Ethics Committee and the Ministry of Health in Punjab, Pakistan. SPSS (SPSS 17.0.1) was used for the descriptive statistical analyses.

## Results

### Descriptive statistics

Of the physicians included in the study, 71.9% were ≤ 30 years of age. Just over half (57.2%) were single and 41.8% were married. In terms of setup, 56.5% were working only in public, 23.5% only in private and 19.8% in both sectors. Mean working hours per week were 56.9. Only 11.4% had post graduate qualifications. Physicians' mean number of work years at their current health facility was 2.5 years.

### Motivators among primary and secondary health care physicians

Thirty separate factors were identified from physicians' responses. Of these, 10 were classified as intrinsic, 16 as organizational and four as socio-cultural factors (Table 1). The six most frequent motivators in current job settings and in general reported by primary and secondary physicians working in the public sector are presented in Table 2. Overall, most of the motivating factors reported in current jobs were intrinsic and socio-cultural. Of these, physicians reported serving people and respect as two of the most important motivators across setups. The organizational factor reported most frequently was good pay. Motivating factors in current jobs were very similar between primary and secondary setups.

**Table 1:** Most frequent motivators and demotivators among physicians

Organizational	Socio-cultural	Intrinsic
***Motivators***		

• Opportunities for higher qualifications	• Respect	• Serving people
• Good working and hygienic conditions	• Social rewards	• Work interest
• Personal safety		• Career growth
• Good professional experience		• Ability to support oneself and family
• Good pay		• Autonomy
• Financial incentives other than pay		• Empowerment

***Demotivators***		

• Less pay	• Disrespect	• Less career growth
• Poor working and hygienic conditions	• Poor interpersonal relations	• Inability to support oneself and family
• Fewer opportunities for higher qualifications	• Less social rewards	
• Less personal safety	• Less personal and social time	
• Heavy work load		
• Long duty hours		
• Resource unavailability		
• Fewer promotion opportunities		
• Poor supervision		

**Table 2:** Motivators among primary and secondary health care physicians

Primary health care physicians				Secondary health care physicians			
**Motivator in current job**	**Responses**	**Frequency (%)**	**Category**	**Motivator in current job**	**Responses**	**Frequency (%)**	**Category**

1. Serving people	26	89.7	Intrinsic	1. Serving people	27	96.4	Intrinsic
2. Respect	22	75.9	Socio-cultural	2. Respect	22	78.6	Socio-cultural
3. Good pay	21	72.4	Organizational	3. Good pay	19	67.9	Organizational
4. Ability to support oneself and family	19	65.5	Intrinsic	4. Ability to support oneself and family	18	64.3	Intrinsic
5. Empowerment	12	41.4	Intrinsic	5. Empowerment	13	46.4	Intrinsic
6. Autonomy	8	27.6	Intrinsic	6. Autonomy	7	25.0	Intrinsic

**Motivator in general perception**	**Responses**	**Frequency (%)**	**Category**	**Motivator in general perception**	**Responses**	**Frequency (%)**	**Category**

1. Serving people	19	63.3	Intrinsic	1. Serving people	19	63.3	Intrinsic
2. Respect	16	53.3	Socio-cultural	2. Respect	16	53.3	Socio-cultural
3. Good pay	14	46.7	Organizational	3. Good pay	14	46.7	Organizational
4. Opportunities for higher qualification	10	33.3	Organizational	4. Opportunities for higher qualification	9	30.0	Organizational
5. Career growth	7	23.3	Intrinsic	5. Career growth	6	20.0	Intrinsic
6. Personal Safety	7	23.3	Organizational	6. Personal Safety	6	20.0	Organizational

n = 30				n = 30			

Serving people, respect and good pay were also listed as general motivators among primary and secondary health care physicians. In addition, organizational factors such as opportunities for higher qualification and personal safety, if not available in physicians' current jobs, were reported more often as general motivators. As with current job motivators, little difference was noted between primary and secondary health facility physicians.

### Demotivators among primary and secondary health care physicians

In contrast, demotivators in current jobs were mostly organizational factors including fewer opportunities for higher qualifications, resource unavailability and poor supervision (Table 3). Physicians in secondary setups reported less pay as a demotivator. Conversely, physicians working in primary care health facilities more often reported poor working conditions as a demotivator.

**Table 3:** Demotivators among primary and secondary health care physicians

Primary health facility physicians				Secondary health facility physicians			
**Demotivator in current Job**	**Responses**	**Frequency (%)**	**Category**	**Demotivator in current job**	**Responses**	**Frequency (%)**	**Category**

1. Fewer opportunities for higher qualifications	15	50.0	Organizational	1. Fewer opportunities for higher qualifications	14	48.3	Organizational
2. Resource unavailability	11	36.7	Organizational	2. Less career growth	10	34.5	Intrinsic
3. Less career growth	10	33.3	Intrinsic	3. Resource unavailability	10	34.5	Organizational
4. Poor supervision	10	33.3	Organizational	4. Poor supervision	10	34.5	Organizational
5. Poor working and hygienic conditions	10	33.3	Organizational	5. Less Pay	9	31.0	Organizational
6. Less personal safety	8	26.7	Organizational	6. Less personal safety	9	31.0	Organizational

**Demotivator in general perception**	**Responses**	**Frequency (%)**	**Category**	**Demotivator in general perception**	**Responses**	**Frequency (%)**	**Category**

1. Less pay	16	55.2	Organizational	1. Less pay	16	57.1%	Organizational
2. Fewer opportunities for higher qualifications	16	55.2	Organizational	2. Fewer opportunities for higher qualifications	16	57.1%	Organizational
3. Inability to support oneself and family	10	34.5	Intrinsic	3. Poor working and hygienic conditions	9	32.1%	Organizational
4. Poor working and hygienic conditions	9	31.0	Organizational	4. Inability to support oneself and family	9	32.1%	Intrinsic
5. Resource unavailability	8	27.6	Organizational	5. Less career growth	8	28.6%	Intrinsic
6. Less career growth	8	27.6	Intrinsic	6. Resource unavailability	8	28.6%	Organizational

n = 30				n = 30			

As with motivators, the demotivating factors for physicians in general were mostly organizational factors including less pay and fewer opportunities for higher qualifications. Organizational factors were largely considered motivators if they were provided but were perceived as demotivators if they were absent. No major differences among physicians working in primary and secondary health facilities were observed in terms of general demotivators.

### Motivators among public and private tertiary health care physicians

Public tertiary health care physicians reported more intrinsic and socio-cultural and fewer organizational factors as motivators than private physicians in their current jobs (Table 4). These results also show that opportunities for higher qualification was important for both groups but in public setups, tertiary care physicians were also motivated by better professional experience. Conversely, physicians in private setups were motivated by the availability of financial incentives other than pay and good working conditions in their current job.

**Table 4:** Motivators among public and private tertiary health care physicians

Public tertiary health facility physicians				Private tertiary health facility physicians			
**Motivator in current job**	**Responses**	**Frequency (%)**	**Category**	**Motivator in current job**	**Responses**	**Frequency (%)**	**Category**

1. Serving people	177	91.7	Intrinsic	1. Serving people	74	85.1	Intrinsic
2. Respect	172	89.1	Socio-cultural	2. Opportunities for higher qualifications	71	81.6	Organizational
3. Opportunities for higher qualifications	127	65.8	Organizational	3. Respect	67	77.0	Socio-cultural
4. Work interest	84	43.5	Intrinsic	4. Financial incentives other than pay	26	29.9	Organizational
5. Good professional experience	70	36.3	Organizational	5. Ability to support oneself and family	23	26.4	Intrinsic
6. Social rewards	44	22.8	Socio-cultural	6. Good working and hygienic conditions	22	25.3	Organizational

**Motivator in general perception**	**Responses**	**Frequency (%)**	**Category**	**Motivator in general perception**	**Responses**	**Frequency (%)**	**Category**

1. Good pay	121	62.1	Organizational	1. Good pay	53	60.9	Organizational
2. Respect	121	62.1	Socio-cultural	2. Serving people	52	59.8	Intrinsic
3. Serving people	105	53.8	Intrinsic	3. Respect	49	56.3	Socio-cultural
4. Good working and hygienic conditions	47	24.1	Organizational	4. Opportunities for higher qualifications	27	31.0	Organizational
5. Career growth	44	22.6	Intrinsic	5. Career growth	22	25.3	Intrinsic
6. Personal Safety	44	22.6	Organizational	6. Good working and hygienic conditions	21	24.1	Organizational

n = 210				n = 90			

The general motivators, good pay, respect, serving people, good working conditions and career growth were common for both public and private health tertiary health care physicians. The only difference observed was that public sector physicians reported personal safety as a motivator rather than opportunities for higher qualification, as reported by those in the private sector.

### Demotivators among public and private tertiary health care physicians

As with those working in primary and secondary health care facilities, current job demotivators for tertiary health care physicians were mostly organizational, although this differed by public and private setup (Table 5). In public setups, tertiary physicians reported long duty hours, less personal safety and heavy workloads as important demotivators compared with those in private setups who listed poor professional experience, less job security and fewer promotion opportunities. Disrespect was also reported more frequently in public tertiary setups whereas less career growth was a more important demotivator in private setups.

**Table 5:** Demotivators among public and private tertiary health care physicians

Public tertiary health facility physicians				Private tertiary health facility physicians			
**Demotivator in current job**	**Responses**	**Frequency (%)**	**Category**	**Demotivator in current job**	**Responses**	**Frequency (%)**	**Category**

1. Less pay	127	65.5	Organizational	1. Less pay	56	65.9	Organizational
2. Long duty hours	78	40.2	Organizational	2. Poor professional experience	45	52.9	Organizational
3. Less personal safety	75	38.7	Organizational	3. Less job security	24	28.2	Organizational
4. Heavy workload	69	35.6	Organizational	4. Fewer promotion opportunities	23	27.1	Organizational
5. Disrespect	63	32.5	Socio-cultural	5. Less career growth	22	25.9	Intrinsic
6. Poor working and hygienic conditions	50	25.8	Organizational	6. Fewer opportunities for higher qualifications	22	25.9	Organizational

**Demotivator in general perception**	**Responses**	**Frequency (%)**	**Category**	**Demotivator in general perception**	**Responses**	**Frequency (%)**	**Category**

1. Less pay	111	58.4	Organizational	1. Less pay	39	47.6	Organizational
2. Disrespect	62	32.6	Socio-cultural	2. Less career growth	27	32.9	Intrinsic
3. Poor working and hygienic conditions	59	31.1	Organizational	3. Poor interpersonal relations	26	31.7	Socio-cultural
4. Less career growth	52	27.4	Intrinsic	4. Disrespect	25	30.5	Socio-cultural
5. Poor interpersonal relations	51	26.8	Socio-cultural	5. Less personal and social time	24	29.3	Socio-cultural
6. Long duty hours	49	25.8	Organizational	6. Less social rewards	23	28.0	Socio-cultural

n = 210				n = 90			

General demotivators included relatively more socio-cultural factors in private setups than in public setups. Less pay, disrespect, less career growth, poor interpersonal relations were common to physicians in both settings. Fewer social rewards were reported as an important socio-cultural demotivator among physicians working in private tertiary facilities. Conversely, the organizational factor long duty hours was only reported in public setups.

### Motivators among male and female physicians

As with physicians working in primary, secondary and tertiary facilities, the current job motivators among male and female physicians were largely intrinsic and socio-cultural (Table 6). Serving people and respect were highly rated current job and general motivators for both. Male physicians also reported opportunities for higher qualifications and career growth as general motivators. However, female physicians reported personal safety and social rewards as more important.

**Table 6:** Motivators among male and female physicians

Male physicians				Female physicians			
**Motivator in current job**	**Responses**	**Frequency (%)**	**Category**	**Motivator in current job**	**Responses**	**Frequency (%)**	**Category**

1. Serving people	151	89.3	Intrinsic	1. Serving people	153	91.1	Intrinsic
2. Respect	149	88.2	Socio-cultural	2. Respect	134	79.8	Socio-cultural
3. Opportunities for higher qualifications	103	60.9	Organizational	3. Opportunities for higher qualifications	95	56.5	Organizational
4. Work interest	68	40.2	Intrinsic	4. Ability to support myself and family	53	31.5	Intrinsic
5. Good professional experience	42	24.9	Organizational	5. Work interest	47	28.0	Intrinsic
6. Ability to support oneself and family	37	21.9	Intrinsic	6. Good professional experience	43	25.6	Organizational

**Motivator in general perception**	**Responses**	**Frequency (%)**	**Category**	**Motivator in general perception**	**Responses**	**Frequency (%)**	**Category**

1. Good pay	106	61.6	Organizational	1. Respect	105	61.8	Socio-cultural
2. Serving people	103	59.9	Intrinsic	2. Good pay	96	56.5	Organizational
3. Respect	97	56.4	Socio-cultural	3. serving people	92	54.1	Intrinsic
4. Opportunities for higher qualifications	49	28.5	Organizational	4. Personal safety	42	24.7	Organizational
5. Career growth	46	26.7	Intrinsic	5. Good working and hygienic conditions	39	22.9	Organizational
6. Good working and hygienic conditions	42	24.4	Organizational	6. Social rewards	38	22.4	Socio-cultural

n = 180				n = 180			

### Demotivators among male and female physicians

Organizational factors again were among the most common demotivators for male and female physicians in their current jobs (Table 7). Male physicians more often reported heavy workloads and fewer promotion opportunities as demotivators. Poor working conditions and less personal and social time were more important among female physicians.

**Table 7:** Demotivators among male and female physicians

Male physicians				Female physicians			
**Demotivator in current job**	**Responses**	**Frequency (%)**	**Category**	**Demotivator in current job**	**Responses**	**Frequency (%)**	**Category**

1. Less pay	110	64.7	Organizational	1. Less pay	88	52.4	Organizational
2. Disrespect	44	25.9	Socio-cultural	2. Less personal safety	53	31.5	Organizational
3. Heavy work load	43	25.3	Organizational	3. Disrespect	47	28.0	Socio-cultural
4. Less personal safety	41	24.1	Organizational	4. Long duty hours	47	28.0	Organizational
5. Fewer promotion opportunities	40	23.5	Organizational	5. Poor working and hygienic conditions	43	25.6	Organizational
6. Long duty hours	38	22.4	Organizational	6. Less personal and social time	41	24.4	Socio-cultural

**Demotivator in general perception**	**Responses**	**Frequency (%)**	**Category**	**Demotivator in general perception**	**Responses**	**Frequency (%)**	**Category**

1. Less pay	100	61.0	Organizational	1. Less pay	82	49.7	Organizational
2. Poor working and hygienic conditions	49	29.9	Organizational	2. Less career growth	50	30.3	Intrinsic
3. Disrespect	45	27.4	Socio-cultural	3. Less personal safety	50	30.3	Organizational
4. Less career growth	45	27.4	Intrinsic	4. Poor working and hygienic conditions	50	30.3	Organizational
5. Less personal and social time	42	25.6	Socio-cultural	5. Poor interpersonal relations	49	29.7	Socio-cultural

n = 180				n = 180			

Less pay and disrespect were common general demotivators for male and female physicians. Male physicians also reported their inability to support themselves and their families and less personal and social time as frequent demotivators. Conversely, female physicians more commonly reported less personal safety and poor interpersonal relations as general demotivators.

## Discussion

### Intrinsic and socio-cultural factors

Overall, physicians reported more intrinsic and socio-cultural factors rather than organizational in their current jobs as motivators. Certain intrinsic and socio-cultural factors such as serving people, respect and opportunities for career growth were nearly universally reported by physicians across all setups. Social rewards such as recognition by employers and communities have been shown to be among the most important motivating factors for health workers [11,28]. Likewise, career growth and development were also identified in previous studies as an important motivator [29-31].

Intrinsic factors like empowerment and autonomy were important motivators among primary and secondary health care physicians. This may be due to staff shortages and a lack of supervision. It may be necessary for physicians in settings with fewer supervisors to act autonomously, which can in and of itself be a motivator [32], Although at the same time, this may limit job performance and frustrate attempts to provide better service.

### Organizational factors

Conversely, the demotivators reported were more organizational, particularly in current job settings. Less pay was the most frequently reported demotivator, a finding also echoed in other studies [31,33,34]. The issue of less pay could also be aggravated by the higher cost of living in urban settings like Lahore. Additionally, as tertiary facilities are exclusively located in urban areas, this may contribute to the migration of physicians from rural to urban areas. Financial incentives may be important determinants of employee motivation but they are undoubtedly only one among several [7,35-37]. Studies have found that money is rarely even the most important motivator [16,28,38]. The effectiveness of performance-related pay in developing country public sector contexts is also a matter of some debate [39]. Pay-for-performance was introduced in Indonesia to provide career development and promote productivity with mixed results [40]. Likewise, another study in Vietnam found that although financial incentives were important, alone they were not likely to improve health worker performance [13]. Other factors like feedback systems [16,36] and target setting processes [36,41] may also be needed. Furthermore, financial incentives alone have been shown not to prevent health workers from migrating, which is a critical aspect of retaining well-trained, motivated staff [4,13]. Moreover, an excessive focus upon financial incentives to motivate individuals in the public sector may even have a number of negative outcomes [42]. Workers may come to see financial rewards as more important than other forms of recognition, such as appreciation by the community or praise from supervisors, or they may feel conflict between their own notion of public sector values and messages about working for financial gain [42]. Therefore, with regard to the issue of financial forms of motivation, it is important to carefully acknowledge both the benefits and limitations of such an approach. Other non-financial ways of improving motivation such as performance appraisals [43,44] and changes to the functioning of the performance measurement system [45] may therefore be just as important to consider.

Another important organizational demotivator was the lack of opportunities for higher qualifications (specialization) in primary and secondary health care facilities, compared to tertiary facilities. This factor was reported more often by younger physicians. In tertiary facilities, specialization opportunities were largely reported as a motivator. Urbanization trends in the region may be due to many factors such as the possibility of a better quality of life, more educational opportunities for children and greater opportunities to attain higher income levels. This issue may also be aggravated by the fact that specialization opportunities are available for physicians only in tertiary teaching hospitals, which are located exclusively in urban areas like Lahore [22]. Thus, the incentive of higher educational opportunities can be a strong motivator both for health care providers and organizations in these setups. Specialization can also secondarily affect physicians' earning capacity and their potential career growth, which was also found to be an important factor in this study.

### Physicians in public and private setups

In private health facilities there were relatively more organizational motivators reported by physicians in their current jobs like financial incentives other than pay and good working and hygienic conditions, which were absent in public setups. Among most public health care physicians, poor working conditions were reported as a common organizational demotivator. Female physicians, in particular, stressed the importance of good working conditions. Possible reasons for this, particularly in public tertiary settings, could be that greater workloads, long duty hours and night shifts in hospitals necessitate the extensive use of facilities such as cafeterias, changing rooms and toilets in these settings. If the facilities are poor, this could greatly impact current job satisfaction and even the quality of care provided to patients [46].

Related to this is the unavailability of resources reported by physicians in primary and secondary setups as a current job and general demotivator. Poor hospital infrastructure and resource unavailability have also been found to be important demotivators in other studies [4,33,47]. Fundamentally, the lack of appropriate resources can compromise health care quality, despite the intentions or abilities of physicians [29,30,48].

### Female and male physicians

Less personal safety was an important demotivator in the public sector, especially among female physicians. Recent security issues in the region and the absence of responses required from mangers and policymakers may also be contributing to this issue. Physicians often risk their own health and safety to treat patients, therefore an additional security issue can be a strong demotivator. Better security measures in private settings like the presence of guards at the doors may explain the absence of this as a demotivator among private tertiary health care physicians (Malik, *pers comm*).

Female physicians also reported less personal and social time as current job demotivator, which may be related to the added household responsibilities expected of them. The general demotivator poor interpersonal relationships could also reflect the lack of support and empowerment opportunities available to female physicians. Only 10% of the female physicians in Pakistan are specialists, primarily in the gynecology and obstetrics fields. Further, the time required to complete specialization may also discourage women, given their other responsibilities, and further reduce their personal and family time.

An additional consideration is that women in developing countries may prefer to visit female physicians due to a variety of traditional and religious factors, which can have ramifications for the achievement of maternal and child health-related Millennium Development Goals (MDGs). Therefore, promoting the motivation of female physicians can assist in the overall improvement of the health system. Addressing these factors specifically can be a critical aspect in retaining and encouraging women in the medical field.

Male physicians reported their inability to support themselves and their families and less personal and social time as important general demotivators. Men often bear the responsibility of financially supporting themselves and their families. As a consequence, male physicians may choose to supplement their incomes with a second job or open a private practice. However, supplemental employment can result in overwork, fatigue and less personal and social time, with fewer rewards, which can negatively affect motivation as well as lead to burnout, physician error and stress [13,28]. Motivation in public tertiary setups was also reported to be aggravated by factors such as less pay, heavy workloads and long duty hours, which may create a vicious cycle in which organizational factors could secondarily affect intrinsic and socio-cultural motivators.

The drain of doctors from Pakistan is becoming a huge challenge in the planning of the workforce, which can at least be partly attributed to fewer opportunities for higher qualifications [48]. According to the WHO, more than 75% of doctors worldwide work in urban areas [2]. Of the medical students graduated annually in Pakistan, half leave the country for the United States and United Kingdom, mainly to acquire higher qualifications and salaries, and many never return [49]. Recent studies have also shown that 11.7% of Pakistan-trained doctors are currently practicing outside of the country, primarily in 4 countries - UK, Canada, Australia and the USA [50]. Over 6000 doctors have left the country during the last five years, according to the Immigration Bureau of Pakistan, although this official figure is believed to be an underestimate [49]. This financial and intellectual competence loss is compounding an already weak health system. For example, the loss of trained health care providers also leads back to the problem of compromised quality of care and hampers health system progression [33,51]. Given the current socio-economical and political context of Pakistan, this problem is expected to continue, which will only contribute to the burden. Therefore, governments should urgently focus on planning and investing in human resource development as well as creating and improving opportunities to retain qualified health personnel and reverse the tide of HCP emigration.

A limitation of this study was the lower representation of physicians working in primary and secondary setups as more physicians work in tertiary setups in urban areas like Lahore. Also, the physicians who participated were mostly younger and early in their career, which may affect the generalizability of these results. Except opportunities for higher qualification, which was reported more by younger physicians in this part of the study, no other significant difference was found between younger and older physicians in terms of motivators and demotivators. Addressing the issue of motivation among younger physicians, however, is particularly valuable for retaining workers and investing in the future of health care in Pakistan. The results of this study sheds light on important motivational factors that can affect whether or not these physicians chose to stay or take advantage of other opportunities abroad. Therefore, more important may be the motivational issues of those physicians facing future health care challenges and reforms.

## Conclusion

This study was first of its kind in the region to investigate physicians' motivation. Motivational determinants showed some important differences and similarities across setups and by gender. The significant motivators in this study were mostly intrinsic and socio-cultural, which are difficult to affect. However, demotivators were largely organizational factors that could also secondarily affect intrinsic and socio-cultural factors. More importantly, these factors can present opportunities for interventions and aid in the creation of new policies and strategies.

Specifically, in public tertiary setups, there is a need to address the issues of pay, working hours and workloads. In public primary and secondary setups, opportunities for higher qualifications, better supervision and adequate resource provision should also be prioritized. Similarly, addressing the problems of less pay, fewer career opportunities, heavy workloads, unsafe environments and poor working conditions are important considerations for both male and female physician motivation.

In addition to the identification of important motivational determinants among physicians in the region, the findings of this study also suggest that many of these factors can be addressed even at local levels. Therefore, promoting local facility changes could improve physicians' overall motivation and subsequently the quality of health care. Given the existing situation in developing countries like Pakistan, it is essential to address physician motivation in order to decrease physician migration, health care worker shortages and minimize the wastage of already limited resources.

Motivation does not remain static and is dependent on many continuously changing factors. The fact that different factors were reported in current job settings and in general also signifies the importance of context. Thus, future studies using exploratory methods may also be needed to better understand the underlying factors eliciting these responses. Finally, longitudinal studies across different setups and cadres should be conducted to monitor the effects of interventions and provide information for effective policy planning.

## Competing interests

The authors declare that they have no competing interests.

## Authors' contributions

AAM designed, conducted, analyzed the study and developed the manuscript. SSY and AS participated in the design and analysis of the results of the study. ZM contributed to the study design and conduction. RS was involved in the design, conduction and analysis of the study. All authors drafted, read and approved the final manuscript.

## Pre-publication history

The pre-publication history for this paper can be accessed here:

http://www.biomedcentral.com/1472-6963/10/201/prepub
